# Toward a decent learning theory: a structural–psychological framework for understanding students' appraisals of learning

**DOI:** 10.3389/fpsyg.2026.1915779

**Published:** 2026-08-31

**Authors:** Bowen Xue, Hong Luo

**Affiliations:** 1 Affiliated Mental Health Center and Hangzhou Seventh People's Hospital, Zhejiang University School of Medicine, Hangzhou, Zhejiang, China; 2 Department of Psychology and Behavioral Sciences, Zhejiang University, Hangzhou, China; 3 Research Center for Mental Health and Humanities, Zhejiang University School of Medicine, Hangzhou, Zhejiang, China

**Keywords:** decent learning theory, dignity in learning, motivational internalization, perceived decent learning, proximal psychological mechanisms, structural educational conditions

## Abstract

Research on learning has often focused on ability, motivation, self-regulation, and performance, but these approaches do not fully explain why students within the same educational system may appraise learning in markedly different ways. To address this gap, this article proposes Decent Learning Theory (DLT) to explain adolescents' perceived decent learning from a structural–psychological perspective. DLT draws on educational psychology, developmental psychology, the sociology of education, intersectionality theory, and the Psychology of Working Theory. It proposes that structural educational conditions and the relational school environment may contribute to perceived decent learning partly through three proximal psychological mechanisms: learning safety, learning belonging, and meaning in learning. Perceived decent learning is defined as students' overall appraisal of whether learning preserves dignity, operates through procedurally fair arrangements, and supports sustainable development. DLT further positions motivational internalization as a downstream mechanism through which perceived decent learning may contribute to behavioral, psychological, and developmental functioning, while allowing for residual direct associations. The theory provides a set of testable propositions and a framework for measurement development, longitudinal and multilevel research, educational practice, and policy.

## Introduction

In recent years, adolescents' learning-related problems have attracted increasing attention in the fields of education and mental health (Mundy et al., 2023). Against a backdrop of recurring pandemics, regional conflicts, and economic fluctuations, learning-related difficulties among adolescents, such as school refusal and school dropout, have become increasingly prominent (Branje and Morris, 2021; Li et al., 2024; Zulaika et al., 2022). Adolescents' learning problems are not driven by a single factor but instead emerge through the dynamic interaction of multiple school-level environmental factors. On the one hand, learning problems are closely associated with students' psychological vulnerabilities, including anxiety and depressive symptoms (Kajastus et al., 2024; Mundy et al., 2023). On the other hand, their occurrence is strongly linked to structural and relational conditions within school contexts, such as academic adjustment, peer relationships, teacher–student interactions, classroom order, and broader school culture (Košir and Jugović, 2025; Valiente et al., 2020; Wong et al., 2021). Over the years, scholars have proposed various theoretical perspectives to explain learning motivation, academic adjustment, and developmental trajectories in education, including Expectancy–Value Theory (Wigfield and Eccles, 2000), Ecological Systems Theory (Bronfenbrenner, 2000), and Constructivist Learning Theory (Narayan et al., 2013). However, these frameworks often address specific aspects of learning, motivation, adjustment, or context rather than organizing them around students' overall appraisal of whether learning occurs under conditions that preserve dignity and support development. Educational system pressure, evaluation systems, teacher–student power relations, and socioeconomic inequality are often treated as background conditions rather than central elements of theory. As concerns about adolescent mental health and academic stress increase, explanations that focus only on individual factors cannot fully capture adolescents' perceptions of learning in real educational settings. There is a need to develop a theoretical framework that integrates structural conditions and subjective experience in order to clarify the developmental meaning of learning in adolescence.

### Decent learning theory

Traditional theories of learning and motivation have made substantial contributions to understanding the psychological mechanisms underlying students' learning behaviors. Over the past several decades, educational psychology research has increasingly focused on individual-level psychological processes, including learning motivation, self-efficacy, interest structures, and self-regulated learning strategies (Hughes and Devine, 2015; Saracho, 2023; Stern, 2017; Urhahne and Wijnia, 2023). Within these theoretical frameworks, learning is typically conceptualized as the outcome of the combined influence of ability, effort, and psychological resources. Although this line of research has generated important insights into differences in learning performance, it has largely relied on internal psychological variables as the primary explanatory pathway (Soudien, 2020).

At the same time, scholars in the fields of sociology of education, critical pedagogy, and social stratification have argued that learning does not occur in a social vacuum. It is embedded in broader social structures and institutional contexts. Educational participation is closely associated with patterns of resource distribution, institutional evaluation mechanisms, and systems of social stratification (Marginson, 2016; Trinidad, 2024). Students' capacity to fully engage in learning depends not only on their levels of motivation or ability but also on the social environments and institutional conditions in which they are situated (Li and Xue, 2023). For students from socioeconomically disadvantaged backgrounds, those positioned at the margins of the education system, or those studying under intense competition and performance evaluation pressures, contextual factors are not secondary influences. They may constitute important conditions associated with how students perceive learning (Allotey et al., 2023; Thiem and Dasgupta, 2022).

Recent structural changes in educational systems have further intensified this issue. As academic competition increases, standardized evaluation systems expand, digital learning environments develop rapidly, and disparities in family educational investment widen, students' learning contexts are undergoing significant transformation (Benítez-Agudelo et al., 2025; Brachtl et al., 2023; Montes, 2024; Pascoe et al., 2020; Tumuheki et al., 2024). Under these conditions, adolescents' perceptions of learning are increasingly associated with institutional demands, time-related pressures, and performance evaluation systems. Frameworks centered solely on individual motivation, self-regulation, or ability differences are no longer sufficient to capture the complexity of how learning is perceived in contemporary educational settings. A theoretical perspective is therefore needed to integrate structural conditions with subjective perception and provide a more comprehensive understanding of adolescent learning processes.

In this regard, the Psychology of Working Theory (PWT) offers a valuable framework for examining the relationship between individual development and social structure (Duffy et al., 2016). PWT emphasizes social class, resource distribution, and freedom of choice as central contextual conditions in developmental experiences. It positions sociocultural context as a core component of theoretical models explaining career development and work experiences, especially among individuals from low socioeconomic backgrounds or marginalized groups (Blustein et al., 2019). Empirical studies further indicate that social class differences significantly influence access to occupational opportunities, experiences of discrimination and marginalization may disrupt developmental pathways, and perceived freedom of choice plays an important role in career decision-making and developmental satisfaction under structural constraints (DeOrtentiis et al., 2022; Duffy et al., 2015; Heilman et al., 2024). These findings demonstrate that developmental experiences cannot be fully understood without considering the broader social structures in which individuals are embedded.

In addition, several research traditions have offered important insights that inform the development of the DLT. Research on learner autonomy and perceived freedom of choice suggests that students' engagement in learning is not merely a passive response to task demands, but is also shaped by whether they experience some degree of choice and room for action (Evans and Boucher, 2015). Work on critical consciousness and social cognition further indicates that students do not simply endure unequal arrangements within educational systems; they also interpret, make sense of, and respond to these structures (Inouye et al., 2023). At the same time, studies on school climate, belonging, and relational support consistently show that learning safety, interaction quality, and adolescents' sense of being accepted matter greatly for academic persistence and psychological wellbeing (Maluenda-Albornoz et al., 2023).

Together, these traditions suggest that students' appraisals of learning emerge from the interplay of structural conditions, relational environments, and psychological processes. Drawing on the structure–psychology integration logic of the Psychology of Working Theory, this article therefore proposes DLT as a preliminary theory for understanding adolescents' appraisals of learning in formal educational contexts. DLT organizes its explanatory pathway around two contextual antecedent domains: structural educational conditions and the relational school environment. Structural educational conditions encompass macro-level, institutional, school, and classroom arrangements concerning access to resources and opportunities, evaluation practices, and sustainable learning rhythms. The relational school environment refers to students' experiences of support, inclusion, recognition, and fair treatment in their interactions with teachers and peers.

DLT proposes that these contextual conditions contribute to perceived decent learning through three proximal psychological mechanisms: learning safety, learning belonging, and meaning in learning. Perceived decent learning refers to students' overall appraisal of whether learning preserves dignity, operates through procedurally fair arrangements, and supports sustainable development. This appraisal may subsequently facilitate motivational internalization and contribute to behavioral, psychological, and developmental functioning. To maintain conceptual parsimony, learning competence, agency, and general psychological need satisfaction are not specified as separate components of the initial model. They are treated as related concepts that may inform specific pathways or future extensions of the theory.

### Core assumptions

*DLT is grounded in six assumptions that define its explanatory focus, construct boundaries, and proposed developmental pathway*.

**1. Learning is a structurally and relationally embedded developmental activity**.

Adolescents' perceptions of learning are associated not only with individual ability, motivation, and self-regulation, but also with the institutional, material, evaluative, and relational conditions under which learning occurs.

**2. Educational conditions are unequally distributed across social positions**.

Students in different socioeconomic, regional, cultural, health, and social positions may have unequal access to educational resources, supportive relationships, fair evaluation, and sustainable learning arrangements.

**3. Contextual conditions are related to learning perceptions through proximal psychological mechanisms**.

DLT proposes that structural educational conditions and the relational school environment contribute to students' perceptions of learning partly through learning safety, learning belonging, and meaning in learning.

**4. Perceived decent learning is a distinct overall appraisal**.

Perceived decent learning refers to students' overall judgment of whether learning preserves dignity, operates through procedurally fair arrangements, and supports sustainable development. It is conceptually distinct from academic performance, learning motivation, and the three proximal psychological mechanisms.

**5. Perceived decent learning may facilitate motivational internalization**.

When students perceive learning as dignity-preserving, procedurally fair, and developmentally sustainable, externally organized learning activities may be more readily integrated into their personal values and developmental goals.

**6. Perceived decent learning may contribute to multiple domains of functioning**.

Through motivational internalization, and potentially through additional direct pathways, perceived decent learning may be associated with students' behavioral, psychological, and developmental functioning. Academic performance may be examined as a distal educational outcome, but it does not define whether learning itself is perceived as decent.

### Theoretical positioning

Existing theories provide important but differently focused explanations of students' learning experiences. Expectancy–Value Theory explains engagement and achievement through students' expectations of success and the subjective value assigned to learning activities (Wigfield and Eccles, 2000). Self-Determination Theory focuses on psychological need satisfaction, autonomous regulation, and the internalization of externally organized activities (Ryan and Deci, 2000, 2020). Ecological Systems Theory situates adolescent development within nested interpersonal, institutional, and sociocultural environments (Bronfenbrenner, 2000). Research on school climate and belonging further demonstrates how safety, support, inclusion, and relational membership shape students' participation and adjustment (Baumeister and Leary, 1995; Korpershoek et al., 2020; Wang and Degol, 2016). Educational equity research examines how differences in resources, opportunities, institutional arrangements, and social positions contribute to unequal educational experiences (Marginson, 2016; Trinidad, 2024).

DLT does not seek to replace these perspectives or suggest that motivation, psychological needs, school climate, belonging, or structural inequality have been absent from educational research. Instead, it addresses a more specific theoretical question: how do structural educational conditions and relational school environments become translated into students' overall appraisal of whether learning preserves dignity, operates through procedurally fair arrangements, and supports sustainable development? Existing theories provide essential explanations of particular components of this process, but these components have rarely been organized around perceived decent learning as the focal appraisal.

Self-Determination Theory is particularly relevant to DLT because it explains how externally organized activities may become integrated into an individual's values and goals (Ryan and Deci, 2000, 2020). DLT draws on this account by positioning motivational internalization as a mechanism following perceived decent learning. However, learning belonging is not treated as interchangeable with relatedness need satisfaction. Learning belonging refers specifically to students' sense that they are accepted and legitimately situated within a learning community, whereas relatedness need satisfaction represents a broader experience of mutual connection and care. Similarly, DLT does not include general psychological need satisfaction as an additional mediator in its initial model. It draws on SDT to explain the downstream motivational process while retaining a distinct focus on students' appraisal of educational conditions.

The Psychology of Working Theory provides the closest structural foundation for DLT. PWT places economic constraints, marginalization, access to opportunity, and psychological processes within a model explaining access to decent work and subsequent functioning (Blustein et al., 2019; Duffy et al., 2016). DLT adapts this structure–psychology logic to educational contexts, but it does not assume that work and learning are equivalent. Adolescents often participate in education under compulsory or highly regulated conditions, have limited freedom to leave or change their learning environments, and depend on schools and teachers for evaluation, recognition, resources, and future opportunities. Learning also occurs during a formative developmental period and is organized through intensive teacher–student and peer relationships. These characteristics require a theory specifically concerned with the dignity and developmental sustainability of learning.

The incremental contribution of DLT lies in integrating these lines of research into a directional structural–psychological pathway. Structural educational conditions and the relational school environment are proposed to shape learning safety, learning belonging, and meaning in learning. These proximal psychological mechanisms contribute to perceived decent learning, which may facilitate motivational internalization and subsequently influence behavioral, psychological, and developmental functioning. Academic performance may be examined as a distal educational outcome, but neither performance nor motivation defines whether learning itself is perceived as decent.

**Figure 1:**
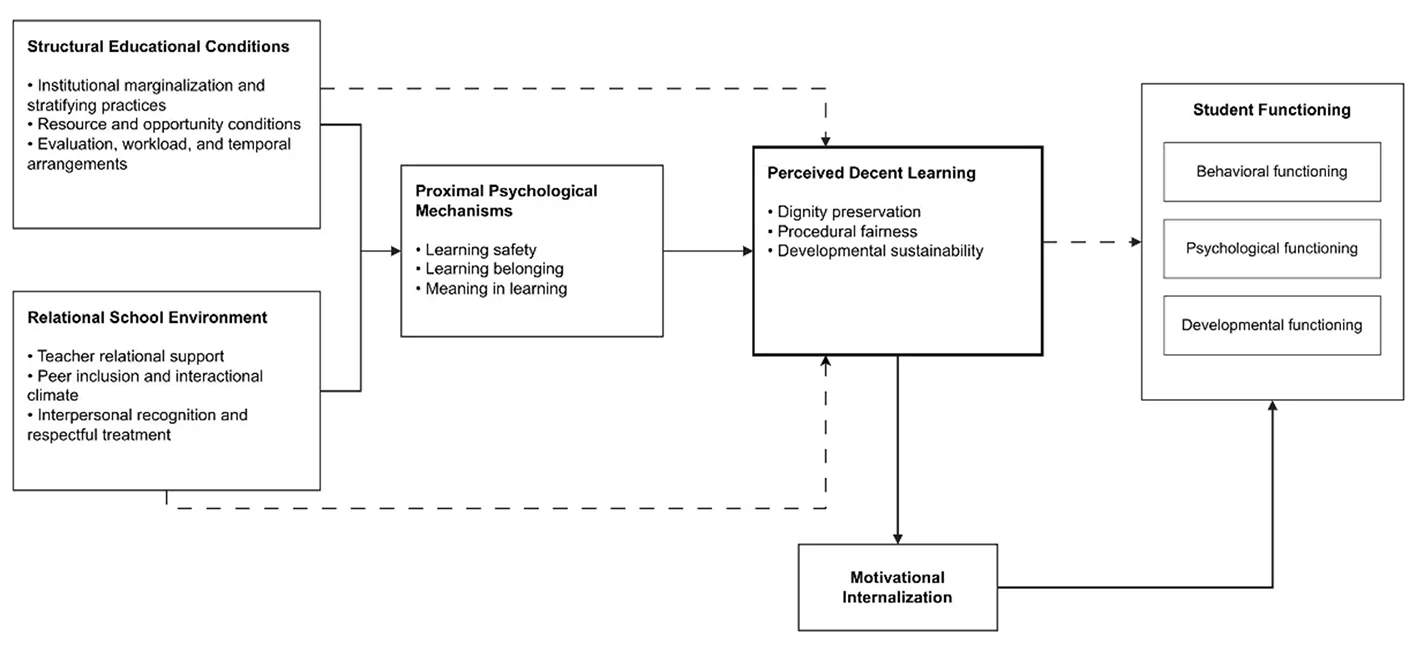
Proposed Conceptual Model of Decent Learning Theory. Solid arrows represent the primary proposed pathways. Dashed arrows represent the residual direct associations specified in Propositions 4 and 8. Developmental stage, social position, cultural context, and educational system are treated as boundary conditions rather than as additional core constructs.

DLT is therefore distinguished by its focal construct and integrative explanatory sequence rather than by the claim that each component of the theory is entirely new. Its central proposition is that the interaction of structural conditions, relational experiences, and proximal psychological processes contributes to students' overall appraisal of whether learning is dignity-preserving, procedurally fair, and developmentally sustainable. This appraisal provides an additional level of explanation between educational conditions and students' motivational and developmental functioning. Figure 1 summarizes the proposed architecture of DLT. Solid arrows represent the primary theoretical sequence from contextual antecedents through proximal psychological mechanisms and perceived decent learning to motivational internalization and student functioning. Dashed arrows indicate the residual direct associations retained in Propositions 4 and 8.

Accordingly, DLT distinguishes between theoretical elements adapted from prior work and propositions that are specific to its present formulation. The emphasis on structural constraints and opportunity conditions is informed primarily by PWT, ecological systems theory, and educational equity research; the relational pathways draw on school-climate and belonging research; and the account of motivational internalization is derived from SDT. DLT does not claim these individual mechanisms as novel. Its more specific contribution is to position perceived decent learning as a distinct appraisal of dignity preservation, procedural fairness, and developmental sustainability and to propose a sequence in which learning safety, learning belonging, and meaning in learning contribute to this appraisal, which in turn precedes motivational internalization and subsequent student functioning. The proposed ordering, the focal position of perceived decent learning, and the retained residual direct pathways therefore represent DLT-specific propositions that require empirical testing.

Table 1 provides a concise overview of the core constructs in DLT, including their definitions, theoretical roles, and principal theoretical sources.

**Table 1:** Core constructs of decent learning theory.

Construct	Concise definition	Role in DLT	Key theoretical sources
Structural educational conditions	Institutional, material, evaluative, temporal, and opportunity arrangements that shape the conditions under which learning occurs.	Contextual antecedent	Bronfenbrenner, 2000; Duffy et al., 2016; Marginson, 2016
Relational school environment	The patterned quality of support, inclusion, recognition, and respectful treatment in students' interactions with teachers and peers.	Contextual antecedent	Wang and Degol, 2016; Korpershoek et al., 2020
Learning safety	Students' sense that they can express ideas, ask questions, attempt unfamiliar tasks, and make mistakes without anticipating humiliation or rejection.	Proximal psychological mechanism	Wansink et al., 2023
Learning belonging	Students' sense that they are accepted and legitimately positioned within a learning community.	Proximal psychological mechanism	Baumeister and Leary, 1995; Allen et al., 2024
Meaning in learning	Students' understanding of learning as coherent, worthwhile, and connected to personally relevant development and future direction.	Proximal psychological mechanism	Martela and Steger, 2016; Novak, 2002
Perceived decent learning	Students' overall appraisal of whether learning preserves dignity, operates through procedurally fair arrangements, and supports sustainable development.	Focal appraisal	Duffy et al., 2016; Blustein et al., 2019; present DLT formulation
Motivational internalization	The process through which externally organized learning becomes personally endorsed and integrated into students' values and developmental goals.	Downstream psychological mechanism	Ryan and Deci, 2000, 2020
Behavioral functioning	Students' expression, engagement, persistence, and exploratory participation in learning activities.	Downstream functioning domain	Schnitzler et al., 2021
Psychological functioning	Students' emotional adjustment and self-evaluation within learning contexts.	Downstream functioning domain	Fidan and Koçak Usluel, 2024
Developmental functioning	Students' longer-term educational goals, commitment, and continued participation in educational pathways.	Downstream functioning domain	Branje, 2022; Branje et al., 2021

### Perceived decent learning as the core appraisal

Perceived decent learning is the focal construct of DLT. It refers to students' overall evaluative judgment of whether learning preserves personal dignity, operates through procedurally fair arrangements, and supports sustainable development. Building on the structure–psychology logic of the Psychology of Working Theory, the construct focuses on the quality of learning as a developmental condition rather than on students' level of achievement, motivation, satisfaction, or adaptation (Blustein et al., 2019; Duffy et al., 2016).

Perceived decent learning is conceptually distinct from both its contextual antecedents and its proximal psychological mechanisms. Structural educational conditions and the relational school environment describe the institutional, material, evaluative, and interpersonal contexts in which learning occurs. Learning safety, learning belonging, and meaning in learning describe the psychological processes through which students interpret and respond to these contexts. Perceived decent learning represents the broader appraisal that may emerge from these processes.

In the initial formulation of DLT, perceived decent learning is organized around three evaluative principles. The first is **dignity preservation**, which refers to whether students perceive themselves as being treated as persons of equal worth rather than as objects of ranking, control, or humiliation. Recognition and respectful treatment are important foundations of personal worth and social dignity in institutional interactions.

The second principle is **procedural fairness**, which concerns whether educational rules, evaluation practices, and access to opportunities are perceived as transparent, consistent, and just. Students are sensitive to the transparency and consistent application of evaluative standards, while constructive and respectful feedback can connect assessment more closely to learning and development.

The third principle is **developmental sustainability**, which refers to whether learning arrangements allow continued growth without chronically undermining rest, psychological functioning, social participation, or longer-term development. Educational workload, extended study time, and performance pressure can reorganize students' everyday time and reduce opportunities for recovery, physical activity, and social connection.

These principles do not require students to perceive every aspect of learning positively. A student may recognize that learning is demanding while still judging it to be fair, respectful, and developmentally sustainable. Conversely, a student may achieve high academic performance while perceiving the learning process as degrading, unfair, or psychologically depleting. Perceived decent learning is therefore neither equivalent to satisfaction nor inferred from academic success.

Perceived decent learning is proposed as a continuous rather than dichotomous appraisal. Students may perceive different degrees of dignity preservation, procedural fairness, and developmental sustainability across learning contexts and over time. The construct may also vary within the same student across subjects, classrooms, schools, or educational transitions.

This narrower definition is intended to preserve the conceptual distinctiveness of perceived decent learning. Learning safety, learning belonging, meaning in learning, supportive relationships, and recognition remain theoretically important, but they are positioned as psychological mechanisms or contextual conditions rather than repeated as defining dimensions of the focal construct. This distinction makes it possible to examine whether these factors predict perceived decent learning and whether perceived decent learning provides explanatory value beyond related constructs such as school climate, academic belonging, psychological safety, learning motivation, and school satisfaction.

### Measurement and research design

The measurement of perceived decent learning should follow the conceptual boundaries specified in DLT. Because the focal construct refers to students' overall appraisal of their learning conditions, perceived decent learning should be assessed primarily through student self-report. Teacher evaluations, classroom observations, and institutional indicators should not be treated as interchangeable indicators of students' perceived decent learning. Instead, they provide information about the contextual conditions in which students' appraisals are formed.

In its initial measurement formulation, perceived decent learning is conceptualized as a multidimensional construct comprising three related but distinct appraisal domains: dignity preservation, procedural fairness, and developmental sustainability. Each domain may be assessed through multiple student-reported items. At this theoretical stage, DLT does not assume either a reflective or formative higher-order specification. Future studies should examine whether the three domains are best represented as correlated dimensions, a higher-order structure, or another multidimensional configuration. The measurement structure should therefore be treated as an empirical question rather than fixed in advance.

The distinction between indicators and antecedents is central to the measurement framework. Student reports of whether they are treated with dignity, whether educational procedures are fair, and whether learning arrangements are developmentally sustainable serve as indicators of perceived decent learning. By contrast, resource allocation, academic workload, evaluation policies, student–teacher ratios, teacher support, peer climate, and access to learning opportunities should be treated as contextual antecedents or external validation variables. Learning safety, learning belonging, and meaning in learning should be measured as separate proximal psychological mechanisms.

Future instrument development should proceed in stages. Initial qualitative studies should examine how students understand dignity, fairness, and developmental sustainability across educational contexts. Item development may then be followed by content validation, exploratory and confirmatory factor analyses, and tests of convergent, discriminant, and incremental validity. Competing measurement models should be compared to determine whether the three appraisal domains are best represented as correlated dimensions, a higher-order structure, or another multidimensional configuration. These analyses should also examine whether perceived decent learning can be distinguished from school climate, school belonging, psychological safety, academic stress, learning motivation, and school satisfaction.

Future empirical tests should distinguish clearly among levels of analysis. Perceived decent learning, learning safety, learning belonging, meaning in learning, motivational internalization, and the three functioning domains are primarily student-level constructs because they concern students' psychological experiences, appraisals, and functioning. Structural educational conditions may be operationalized at the school or system level through indicators such as resource allocation, evaluation policies, workload arrangements, and opportunity structures. Relational school conditions may be assessed either as student-level perceptions of teacher and peer interactions or, when appropriately aggregated, as classroom- or school-level relational characteristics.

Because students are nested within classrooms and schools, multilevel models will often be appropriate for testing cross-level relationships between contextual conditions and student-level psychological processes. Longitudinal designs are also needed to examine whether contextual conditions precede changes in the three proximal mechanisms, perceived decent learning, motivational internalization, and subsequent functioning. Multi-informant data may strengthen contextual assessment, but they should supplement rather than replace students' own reports of perceived decent learning.

### Theoretical propositions of decent learning theory

DLT specifies a directional structural–psychological pathway linking educational conditions with students' perceptions of learning and subsequent functioning. The propositions are organized into four stages: contextual conditions and proximal psychological mechanisms, the formation of perceived decent learning, motivational internalization, and behavioral, psychological, and developmental functioning. These propositions describe expected relationships that remain open to empirical confirmation, refinement, or rejection.

#### Proposition 1a

More favorable structural educational conditions, including greater opportunity accessibility, lower exposure to marginalization, fairer institutional arrangements, and more sustainable learning demands, will be positively associated with learning safety, learning belonging, and meaning in learning.

#### Proposition 1b

A more supportive relational school environment, characterized by teacher support, peer inclusion, recognition, and fair interpersonal treatment, will be positively associated with learning safety, learning belonging, and meaning in learning.

#### Proposition 2

Learning safety, learning belonging, and meaning in learning will each be positively associated with perceived decent learning.

#### Proposition 3

Learning safety, learning belonging, and meaning in learning will mediate the relationships between structural educational conditions, the relational school environment, and perceived decent learning.

#### Proposition 4

Structural educational conditions and the relational school environment may retain direct associations with perceived decent learning after the three proximal psychological mechanisms are considered.

#### Proposition 5

Perceived decent learning will be positively associated with motivational internalization, such that students who perceive learning as dignity-preserving, procedurally fair, and developmentally sustainable will be more likely to integrate learning into their personal values and developmental goals.

#### Proposition 6

Motivational internalization will be positively associated with behavioral, psychological, and developmental functioning.

#### Proposition 7

Motivational internalization will mediate the relationships between perceived decent learning and behavioral, psychological, and developmental functioning.

#### Proposition 8

Perceived decent learning may retain direct associations with behavioral, psychological, and developmental functioning after motivational internalization is considered.

These propositions constitute the initial testable structure of DLT. They do not assume that the proposed pathways are universal or invariant across educational settings. Their magnitude and form may vary across developmental stages, educational systems, social positions, and cultural contexts. Future research should therefore examine both the central pathways and the conditions that define the explanatory boundaries of the theory.

### Contextual antecedents of perceived decent learning

DLT organizes the contextual antecedents of perceived decent learning into two broad domains: structural educational conditions and the relational school environment. Structural educational conditions refer to the institutional, material, evaluative, and opportunity arrangements that shape the conditions under which learning occurs. The relational school environment refers to the quality of support, inclusion, recognition, and interpersonal treatment that students experience in their interactions with teachers and peers. The distinction between these domains is analytical rather than absolute because institutional arrangements influence everyday relationships, while repeated relational practices may reinforce or challenge institutional patterns.

Social position is relevant to DLT, but it is not specified as a third domain of antecedents. An intersectional perspective suggests that socioeconomic position, regional location, gender, ethnicity, migration background, and health or disability status may jointly shape students' exposure to educational resources, evaluative arrangements, marginalization, and supportive relationships (Cole, 2009; Gkiouleka et al., 2018). These positions should therefore be understood as distal conditions that influence how structural and relational antecedents are distributed and experienced.

This distinction prevents social identities from being treated as inherent sources of educational disadvantage. DLT proposes that unequal perceptions of learning emerge through the institutional and relational conditions associated with particular social positions rather than from those identities themselves. For example, socioeconomic disadvantage may affect perceived decent learning through restricted access to learning opportunities, while disability status may become relevant through inaccessible arrangements, stigmatizing evaluation, or exclusionary interactions.

Social positions may also function as boundary conditions in future empirical tests. The strength or form of the proposed pathways may vary across combinations of socioeconomic, regional, cultural, migration, gender, and health-related positions. However, the initial DLT model remains focused on the two proximal contextual domains through which such inequalities become educationally and psychologically consequential.

### Structural educational conditions

Structural educational conditions refer to macro-level or institutional features of schooling, such as school organization, resource allocation, and opportunity structures, that shape the learning environment prior to students' entry into specific interactions (Astor et al., 2021; Hanselman et al., 2021; Mejía-Rodríguez and Kyriakides, 2022; Zengilowski et al., 2023). The DLT argues that these conditions constitute an essential structural foundation for whether learning can be experienced as decent.

### Institutional marginalization and stratifying practices

Institutional marginalization refers to school-level arrangements and routine practices that systematically limit some students' visibility, voice, participation, or access to valued educational opportunities. Unlike interpersonal exclusion, which occurs through teacher–student or peer interactions, institutional marginalization is embedded in the rules and organizational practices through which schools classify students, allocate resources, regulate participation, and distribute educational opportunities (Mowat, 2015; Tarabini et al., 2018).

Institutional marginalization may operate through academic tracking, performance-based grouping, public ranking, disciplinary systems, inaccessible learning arrangements, or the repeated use of labels that assign students a relatively fixed position within the school hierarchy (Jain et al., 2024; Terrin and Triventi, 2023). Although such practices may be justified as mechanisms for organizing instruction or evaluation, they can produce enduring differences in status and opportunity. Students placed in low-expectation categories may receive fewer developmental opportunities, less academic investment, and reduced access to valued forms of participation.

Evaluation and surveillance practices may further reinforce these inequalities. Persistent comparison, public ranking, and highly performance-oriented monitoring can position students as objects of judgment rather than participants in development (Pérez-Jorge et al., 2025). When institutional evaluation primarily communicates deficiency or fixed status, students may find it more difficult to regard educational procedures as fair or to view learning as a credible pathway for growth.

Within DLT, institutional marginalization is treated as a structural antecedent rather than as a psychological state or interpersonal experience. It is expected to influence perceived decent learning partly by undermining learning safety, learning belonging, and meaning in learning. Teacher- and peer-based forms of exclusion are addressed separately within the relational school environment because they operate through everyday interpersonal interactions rather than through institutional arrangements alone.

### Resource and opportunity conditions

Resource and opportunity conditions refer to the material, curricular, temporal, and institutional arrangements that shape what learning opportunities are available to students and the conditions under which those opportunities can be pursued. They include access to instructional support, curricular choice, digital resources, enrichment activities, academic guidance, and other educational opportunities distributed across family, school, and community contexts (Qin et al., 2025; Roscigno et al., 2006).

Unequal access to educational resources can narrow the range of learning activities in which students are able to participate. Students in better-resourced settings may encounter broader curricular options, sustained instructional support, and more varied opportunities for exploration, whereas students in resource-constrained settings may experience more restricted learning pathways and fewer opportunities for development (Brännlund and Edlund, 2020; Decker et al., 2025; Neugebauer and Prediger, 2023). Within DLT, these differences are expected to influence students' learning safety, learning belonging, and meaning in learning by shaping whether they can participate, receive support, and connect learning with future development.

The temporal organization of learning also constitutes a structural condition. Extended study hours, intensive homework requirements, and continuous performance monitoring may displace time for rest, physical activity, social participation, and psychological recovery (Cashman et al., 2023; Jacobsen, 2025; Liu et al., 2023; Ran et al., 2025). DLT distinguishes these objective or institutionally organized demands from students' appraisal of developmental sustainability. Workload and time arrangements are contextual antecedents, whereas the judgment that learning can be sustained without chronic psychological or developmental depletion forms part of perceived decent learning.

Opportunity accessibility should likewise be distinguished from students' subjective interpretation of opportunity. Objective access to courses, guidance, digital resources, enrichment activities, and educational pathways belongs to the structural context. Students' interpretations of whether learning is connected to attainable and worthwhile development are more closely related to meaning in learning. Perceived opportunity is therefore not specified as an additional psychological mediator in the initial DLT model, although future studies may examine whether it adds explanatory value.

Resource constraints may coexist with institutional marginalization. Limited access to resources can restrict participation and increase students' peripheral position within educational activities, while institutional labeling and stratifying practices may further reduce access to instructional support and valued opportunities (Hamel et al., 2022; Nungu et al., 2023; Prananto et al., 2025). These conditions are analytically distinguishable but may reinforce one another in practice.

Within DLT, resource and opportunity conditions are treated as contextual antecedents rather than as indicators of perceived decent learning. Their effects are expected to operate primarily through learning safety, learning belonging, and meaning in learning, although residual direct associations with perceived decent learning may also remain.

### Relational school environment

The relational school environment refers to the patterned quality of students' everyday interactions with teachers and peers. It includes the availability of support, opportunities for participation, interpersonal inclusion, recognition, and respectful treatment within school-based relationships. These relational conditions are conceptually external to students' psychological states. They may contribute to learning safety, learning belonging, and meaning in learning, but they are not interchangeable with these mechanisms.

DLT distinguishes relational experiences from both institutional arrangements and students' broader appraisals. Formal evaluation rules, ranking systems, disciplinary procedures, and opportunity allocation belong to structural educational conditions. The interpersonal manner in which teachers communicate expectations, respond to mistakes, and deliver feedback belongs to the relational school environment. Students' overall judgment of whether educational procedures are fair belongs to perceived decent learning.

### Teacher relational support

Teacher relational support refers to the availability of responsive guidance, encouragement, constructive feedback, and respectful interaction within teacher–student relationships. Teachers communicate not only academic information but also messages about whether students' contributions, difficulties, and potential are taken seriously (Laranjeira and Teixeira, 2024; Semeraro et al., 2020). Supportive teacher interactions may reduce the interpersonal risks associated with asking questions, expressing unfinished ideas, and making mistakes. When teachers respond constructively rather than through ridicule, public comparison, or dismissal, students are more likely to experience the classroom as a setting in which participation is possible and worthwhile (Gonda et al., 2024). Within DLT, teacher relational support is therefore expected to contribute to learning safety and meaning in learning, which may subsequently strengthen perceived decent learning.

### Peer inclusion and interactional climate

Peer inclusion and interactional climate refer to the extent to which peer relationships permit participation, acceptance, collaboration, and expression without persistent ridicule, isolation, or exclusion. During adolescence, peer networks provide an important social context through which students interpret classroom participation and learning activities (Wang and Eccles, 2012). Inclusive peer climates may provide students with opportunities to contribute to shared tasks and participate in classroom interaction. By contrast, exclusionary group boundaries, hostile peer norms, and ridicule may restrict participation and increase the perceived interpersonal risks of learning (Hamel et al., 2022; Kilday and Ryan, 2025; Seo et al., 2025). DLT treats these peer conditions as antecedents of learning belonging rather than as learning belonging itself. Learning belonging refers to the psychological sense of legitimate membership that may develop from repeated experiences of inclusion and participation.

### Interpersonal recognition and respectful treatment

Interpersonal recognition refers to whether students' efforts, progress, viewpoints, and developmental potential are acknowledged within everyday school interactions. Recognition communicates that students are valued participants whose contributions are worthy of attention, while persistent disregard or devaluation may weaken their position within the learning community (Rothers and Cohrs, 2023; Wentzel et al., 2017).

Respectful treatment is especially relevant during evaluation and feedback. Formal assessment criteria belong to the structural context, but feedback is delivered through interpersonal interaction. Feedback that acknowledges effort, explains areas for development, and avoids humiliation may support students' understanding of learning as a legitimate developmental activity (Harrison et al., 2016; Wang and Hazari, 2018). Within DLT, interpersonal recognition and respectful treatment are expected to contribute primarily to learning belonging and meaning in learning and may also have a direct association with perceived decent learning.

Taken together, teacher relational support, peer inclusion, and interpersonal recognition constitute the relational school environment in the initial DLT model. These conditions are proposed to shape perceived decent learning primarily through learning safety, learning belonging, and meaning in learning. Residual direct relationships may also remain because some relational experiences, particularly repeated disrespect or recognition, may contribute directly to students' appraisal of whether learning preserves dignity.

### Proximal psychological mechanisms

DLT proposes that structural educational conditions and the relational school environment are translated into students' appraisals of learning partly through three proximal psychological mechanisms: learning safety, learning belonging, and meaning in learning. These mechanisms describe students' immediate psychological responses to educational and relational contexts rather than objective features of those contexts.

Learning safety concerns whether students feel able to express ideas, attempt unfamiliar tasks, and make mistakes without anticipating humiliation or rejection (Wansink et al., 2023). Learning belonging concerns whether students experience themselves as accepted and legitimately positioned within a learning community (Allen et al., 2024; Baumeister and Leary, 1995). Meaning in learning concerns whether students understand learning as personally valuable, coherent, and connected to development (Martela and Steger, 2016). Although these mechanisms may be related, each captures a theoretically distinct aspect of how students interpret learning conditions.

The three mechanisms are also distinct from perceived decent learning. They describe specific psychological experiences through which contextual conditions may be interpreted, whereas perceived decent learning represents the broader appraisal of whether learning preserves dignity, operates through procedurally fair arrangements, and supports sustainable development. For example, supportive peer interactions may strengthen learning belonging, which may subsequently contribute to a more favorable appraisal of learning, but peer support, belonging, and perceived decent learning remain separate constructs.

DLT initially proposes parallel indirect pathways through learning safety, learning belonging, and meaning in learning. The theory does not assume that all contextual influences are fully mediated by these mechanisms. Structural educational conditions and the relational school environment may also retain direct associations with perceived decent learning, particularly when students directly evaluate institutional fairness, respectful treatment, or unsustainable learning arrangements. The relative strength of the three indirect pathways and any residual direct relationships should be examined empirically.

### Learning safety

Learning safety refers to students' psychological sense that they can express ideas, ask questions, attempt unfamiliar tasks, make mistakes, and reveal incomplete understanding without anticipating humiliation, ridicule, rejection, or other negative interpersonal consequences. It is a domain-specific psychological experience within learning situations rather than an objective characteristic of the classroom or a general judgment about whether learning is decent.

Learning activities frequently require students to expose uncertainty in front of teachers and peers. Answering questions, participating in discussion, and attempting unfamiliar tasks may therefore involve interpersonal risk. When students anticipate negative judgment or embarrassment, they may remain silent, avoid challenge, or reduce their involvement in classroom activities (Wansink et al., 2023; Grieve et al., 2021). By contrast, constructive responses to mistakes and openness to different viewpoints can reduce anticipated interpersonal threat and make exploratory participation more psychologically possible (Wansink et al., 2023).

Within DLT, learning safety may be shaped by both structural educational conditions and the relational school environment. Institutional practices such as public ranking, punitive evaluation, or intensive surveillance may increase students' perceived exposure to judgment. Supportive teacher responses and inclusive peer interactions may reduce this risk. These institutional and relational conditions remain external antecedents, whereas learning safety refers to the psychological experience that develops in response to them.

Learning safety is proposed to contribute to perceived decent learning, particularly by informing students' broader appraisal of whether participation in learning preserves their dignity. However, learning safety is not itself equivalent to perceived decent learning. A student may feel able to express ideas in a particular classroom while still judging the evaluation system to be unfair or the overall learning demands to be developmentally unsustainable. This distinction permits learning safety to be examined as a mediator rather than being repeated as a defining dimension of perceived decent learning.

### Learning belonging

Learning belonging refers to students' psychological sense that they are accepted and legitimately positioned as participants within a learning community. It concerns whether students experience themselves as having a recognized place in classroom activities, collaborative learning, and other shared educational practices rather than as peripheral or excluded participants (Allen et al., 2024; Do et al., 2024).

Learning belonging is related to, but distinct from, the relatedness need described in Self-Determination Theory. Relatedness need satisfaction concerns a broader experience of connection, mutual care, and interpersonal support across relationships (Ryan and Deci, 2000, 2020). Learning belonging is more context-specific. It concerns students' perceived membership and legitimacy within communities organized around learning. A student may experience supportive relationships with particular teachers or peers while still feeling marginal to classroom participation or academic interaction.

Learning belonging is also distinct from peer inclusion and teacher relational support. Peer inclusion, supportive interaction, and interpersonal recognition are relational antecedents that may contribute to belonging. Learning belonging refers to the psychological experience that may develop through repeated opportunities to participate, contribute, and receive acknowledgment within the learning community. Conversely, exclusionary peer norms, institutional labeling, or limited opportunities for participation may weaken this sense of membership (Baumeister and Leary, 1995; Korpershoek et al., 2020).

Within DLT, learning belonging is proposed to contribute to perceived decent learning by informing whether students experience learning as a form of legitimate and valued participation. However, it is not equivalent to perceived decent learning. Students may feel accepted within their peer group while still judging educational procedures to be unfair or learning demands to be developmentally unsustainable. This distinction allows learning belonging to operate as a proximal psychological mechanism rather than as a defining dimension of the core appraisal.

### Meaning in learning

Meaning in learning refers to students' psychological understanding of learning as coherent, worthwhile, and connected to personally relevant development and future direction. It concerns whether students can identify why learning matters and how particular learning activities relate to growth, capability development, or longer-term goals (Howard et al., 2021; Martela and Steger, 2016; Novak, 2002).

Meaning in learning may be shaped by both structural and relational conditions. Access to varied learning opportunities may make it easier for students to connect educational activities with future possibilities, while constructive guidance and recognition may help them understand the developmental significance of their efforts. By contrast, highly repetitive, inaccessible, or exclusively performance-oriented learning arrangements may make learning appear disconnected from personal development.

Meaning in learning is distinct from recognition, procedural fairness, and developmental sustainability. Recognition and respectful treatment are relational conditions, while procedural fairness and developmental sustainability form part of the broader appraisal of perceived decent learning. Meaning in learning refers specifically to the subjective interpretation that learning has value, direction, and developmental significance.

Meaning in learning is also distinct from motivational internalization. Meaning concerns students' understanding of why learning is valuable, whereas motivational internalization concerns the regulatory process through which externally organized learning becomes personally endorsed and integrated into students' values and goals. Students may recognize that learning is useful or developmentally important while still participating primarily because of external requirements. Meaning may therefore support the formation of perceived decent learning without being equivalent to the subsequent internalization of learning motivation.

Within DLT, meaning in learning is proposed as a proximal psychological mechanism contributing to perceived decent learning. When students understand learning as worthwhile and developmentally relevant, they may be more likely to judge the overall learning process as supportive of human development. However, meaning alone is insufficient to establish perceived decent learning. Learning may appear meaningful while still being experienced as unfair, degrading, or developmentally unsustainable.

### Motivational internalization as a downstream mechanism

Motivational internalization refers to the process through which externally organized learning activities become increasingly understood and endorsed as personally relevant and consistent with students' values and developmental goals. Within Self-Determination Theory, internalization describes the gradual transformation of externally regulated activities into more autonomous forms of behavioral regulation. It does not imply that curricular requirements, evaluation demands, or other external structures disappear (Ryan and Deci, 2000, 2020).

Motivational internalization is particularly relevant to adolescent learning because many educational activities are initially organized through curricula, examinations, school rules, and adult expectations. Students may initially participate because learning is required, but externally structured activities can become more personally endorsed when students connect them with their own values, goals, and developing sense of self (Cook and Artino, 2016; Jang, 2008).

Within DLT, motivational internalization is positioned downstream from perceived decent learning. It is not one of the three proximal psychological mechanisms and is not specified as a moderator in the initial model. DLT proposes that students may be more likely to endorse the value of learning when they appraise the learning process as dignity-preserving, procedurally fair, and developmentally sustainable. This proposed direction remains subject to empirical testing.

Motivational internalization is related to, but distinct from, meaning in learning. Meaning in learning concerns whether students understand why learning is worthwhile and developmentally relevant. Motivational internalization concerns the extent to which this value becomes incorporated into students' self-regulation and personally endorsed goals. Students may recognize that learning is useful while continuing to participate mainly because of external pressure. Meaning therefore does not necessarily imply internalization.

DLT proposes that motivational internalization may partially mediate the relationships between perceived decent learning and behavioral, psychological, and developmental functioning. Greater internalization may support more sustained participation, more adaptive psychological functioning, and stronger commitment to longer-term educational pathways. However, the theory does not assume complete mediation. Perceived decent learning may also retain direct associations with these functioning domains.

Future longitudinal research should examine whether perceived decent learning precedes changes in motivational internalization and whether internalization subsequently predicts changes in functioning. Competing temporal models should also be considered because students' motivation and functioning may, in turn, influence how they appraise learning conditions.

### Behavioral, psychological, and developmental functioning

DLT proposes that perceived decent learning may be associated with three downstream domains of student functioning: behavioral functioning, psychological functioning, and developmental functioning. These domains represent theoretically proposed consequences of students' appraisals of learning rather than defining dimensions or indicators of perceived decent learning.

Behavioral functioning concerns how students participate in learning activities, including expression, sustained engagement, persistence, and exploratory participation. Psychological functioning concerns students' emotional experience, self-evaluation, and adjustment within learning contexts. Developmental functioning concerns students' longer-term orientation toward educational pathways, future goals, and continued participation in education.

Perceived decent learning may contribute to these domains through both direct and indirect pathways. When students appraise learning as dignity-preserving, procedurally fair, and developmentally sustainable, they may be more able to participate without persistent self-protection, maintain healthier psychological adjustment, and connect education with longer-term development. These associations may be partly explained by motivational internalization, through which learning becomes more personally endorsed and integrated into students' values and goals.

DLT does not assume that motivational internalization fully accounts for all relationships between perceived decent learning and functioning. Some direct associations may remain, particularly for immediate emotional responses, participation, and adjustment within learning settings. The relative importance of direct and indirect pathways should be examined using longitudinal and multilevel research designs.

Academic performance may also be examined as a distal educational outcome, but it is not treated as a defining indicator of perceived decent learning or as one of the three primary functioning domains in the initial model. This distinction allows future studies to test whether perceived decent learning explains student functioning and academic outcomes beyond conventional measures of motivation, school satisfaction, and achievement.

### Behavioral functioning

Behavioral functioning refers to students' observable participation and sustained involvement in learning activities. In the initial DLT model, it includes three related forms of behavior: classroom expression and interactive participation, task engagement and persistence, and exploratory or challenge-oriented behavior. These behaviors are treated as proposed downstream outcomes rather than as defining dimensions or direct indicators of perceived decent learning.

Classroom expression and interactive participation include asking questions, contributing ideas, participating in discussion, and engaging in collaborative knowledge construction (Chin, 2006; van Balen et al., 2022). Task engagement and persistence concern sustained attention, effort, task completion, and continued involvement when difficulties arise (Abren et al., 2025; Schnitzler et al., 2021). Exploratory and challenge-oriented behavior includes trying unfamiliar strategies, engaging with demanding tasks, and actively seeking additional learning opportunities (García-Almeida and Cabrera-Nuez, 2020; Jia and Cheng, 2024).

DLT proposes that students who appraise learning as dignity-preserving, procedurally fair, and developmentally sustainable may be more likely to participate actively and sustain their involvement. These relationships may be partly explained by motivational internalization because personally endorsed learning is more likely to support persistent and self-directed participation. Perceived decent learning may also retain direct associations with behavior, particularly with students' willingness to express themselves and remain involved in immediate learning situations.

Behavioral functioning is conceptually distinct from learning safety, meaning in learning, and motivational internalization. Learning safety concerns anticipated interpersonal risk, meaning in learning concerns the perceived value and direction of learning, and motivational internalization concerns personally endorsed regulation. Behavioral functioning concerns what students do within learning activities. Agency is therefore not introduced as an additional construct in the initial model, even though initiative may be reflected in exploratory behavior.

The three behavioral forms should also be distinguished empirically. Students may complete assigned tasks consistently without expressing ideas or seeking challenge, while others may participate actively but show limited persistence. Future studies should therefore examine whether classroom participation, task persistence, and exploratory behavior form distinguishable first-order components of a broader behavioral functioning domain and whether they are differentially associated with perceived decent learning and motivational internalization.

### Psychological functioning

Psychological functioning refers to students' emotional and self-evaluative adjustment within learning contexts. In the initial DLT model, it includes two related aspects: learning-related emotional well-being and distress, and students' evaluation of themselves as learners. These experiences are treated as proposed downstream outcomes rather than as dimensions or indicators of perceived decent learning.

Learning-related emotional functioning concerns the relative presence of emotions such as interest, calmness, and enjoyment and the relative absence of persistent anxiety, shame, tension, or stress during educational participation. Emotional experiences can influence students' information processing, engagement, and willingness to remain involved in learning activities (Fidan and Koçak Usluel, 2024). Environments characterized by humiliation, exclusion, or intensive evaluative threat have been associated with less adaptive emotional experiences among students (Fernández-Sogorb et al., 2021; Pérez-Jorge et al., 2025).

Self-evaluative functioning concerns how students understand their worth, capability for development, and legitimacy as learners. Repeated devaluation or negative judgment may contribute to unfavorable learner self-evaluations, whereas credible opportunities for improvement may support academic confidence and more adaptive adjustment to temporary difficulty or failure (Chen et al., 2023; Downing et al., 2020). In DLT, these self-evaluations are psychological outcomes and do not constitute a separate learning competence mechanism.

Psychological functioning is conceptually distinct from learning safety. Learning safety concerns anticipated interpersonal risk when students express uncertainty, participate, or make mistakes. Psychological functioning concerns broader emotional adjustment and self-evaluation that may develop across repeated learning experiences. It is also distinct from perceived decent learning, which concerns students' appraisal of whether learning preserves dignity, operates through procedurally fair arrangements, and supports sustainable development.

DLT proposes that perceived decent learning may be associated with more adaptive psychological functioning both directly and indirectly through motivational internalization. Direct associations may be particularly relevant to immediate emotional responses and students' sense of themselves as learners, whereas internalization may support more sustained adjustment by connecting learning with personally endorsed values and goals. These temporal and mediating relationships require longitudinal testing and should not be interpreted as established causal effects.

### Developmental functioning

Developmental functioning refers to students' longer-term orientation toward education and personal development. In the initial DLT model, it includes future-oriented goal construction, sustained commitment to educational pathways, and continued participation in education over time. These outcomes extend beyond students' immediate behavior or emotional state in a particular learning situation.

Adolescence is an important period for identity development, future planning, and the construction of possible educational and occupational pathways (Branje, 2022; Branje et al., 2021). Students' learning experiences may contribute to how they imagine their future selves and connect present educational participation with longer-term goals (Beal and Crockett, 2010; King et al., 2015). Developmental functioning may therefore be reflected in the clarity and stability of educational goals and in students' capacity to maintain a future-oriented direction when difficulties arise.

Educational persistence represents a related but distinct aspect of developmental functioning. It concerns sustained commitment to schooling, training, or other educational pathways across time rather than persistence in completing a particular task. Possible indicators include intentions to continue education, sustained investment in an educational pathway, continued enrollment, and lower levels of prolonged educational disengagement (Ansari et al., 2020; Li and Xue, 2023). School refusal or withdrawal may be examined as distal indicators, but they should not be interpreted as consequences of perceived decent learning alone because they are shaped by multiple individual, family, and institutional factors.

Developmental functioning is distinct from meaning in learning and motivational internalization. Meaning in learning concerns whether students understand learning as worthwhile and developmentally relevant. Motivational internalization concerns whether learning values become personally endorsed and incorporated into self-regulation. Developmental functioning concerns whether these processes are subsequently reflected in longer-term goals, educational commitment, and sustained participation.

DLT proposes that perceived decent learning may be associated with developmental functioning both directly and indirectly through motivational internalization. Students who appraise learning as dignity-preserving, procedurally fair, and developmentally sustainable may be more likely to regard education as a credible pathway for future development. Motivational internalization may further support this relationship by integrating learning with personally endorsed goals. These proposed temporal relationships require longitudinal examination and should not be interpreted as established causal effects.

## Discussion

The present article makes three main theoretical contributions. First, it introduces perceived decent learning as a distinct overall appraisal organized around dignity preservation, procedural fairness, and developmental sustainability. Second, it specifies a directional pathway through which structural and relational conditions may shape this appraisal through three proximal psychological mechanisms. Third, it distinguishes the formation of perceived decent learning from the downstream process of motivational internalization and proposes direct and indirect relationships with three domains of student functioning.

### Theoretical contributions

The first contribution of DLT is the introduction of perceived decent learning as a focal appraisal construct. Existing research has examined school climate, psychological safety, belonging, meaning in learning, academic stress, educational fairness, and student motivation. These constructs provide important explanations of particular aspects of students' experiences, but they do not necessarily capture students' broader judgment of whether the learning process preserves personal dignity, operates through fair procedures, and supports continued development. Perceived decent learning is therefore not intended as another label for positive school climate, school satisfaction, or academic adjustment. It represents an additional evaluative level through which students interpret the overall quality of learning as a developmental process.

The second contribution is the specification of a cross-level explanatory sequence. Ecological Systems Theory and educational equity research demonstrate that development and educational participation are embedded in institutional, social, and material contexts (Bronfenbrenner, 2000; Marginson, 2016; Trinidad, 2024). School climate and relational research further identify the importance of support, inclusion, safety, and interpersonal treatment (Wang and Degol, 2016). DLT builds on these traditions by proposing how structural educational conditions and the relational school environment may be psychologically translated into an overall appraisal of learning. Learning safety, learning belonging, and meaning in learning are positioned as distinct proximal mechanisms rather than as interchangeable characteristics of the environment or dimensions of perceived decent learning.

The third contribution concerns the relationship between appraisal and motivation. Self-Determination Theory explains how externally organized activities may become increasingly internalized and personally endorsed (Ryan and Deci, 2000, 2020). DLT adopts this account of motivational internalization but places it downstream from perceived decent learning. The theory proposes that students may be more likely to internalize learning values when they judge the learning process to be dignity-preserving, procedurally fair, and developmentally sustainable. This sequence differs from models that position psychological need satisfaction, belonging, meaning, and internalization as parallel predictors of engagement. In DLT, the three proximal mechanisms contribute to the formation of the core appraisal, whereas motivational internalization provides a possible pathway from that appraisal to subsequent functioning.

PWT provides the closest structural foundation for this sequence because it links contextual constraints, psychological processes, access to decent work, and subsequent functioning (Blustein et al., 2019; Duffy et al., 2016). DLT adapts this structural–psychological logic to formal education but does not treat work and learning as equivalent. Adolescents often participate in education under compulsory or highly regulated conditions. They have limited ability to leave or change learning environments and depend on educational institutions for evaluation, recognition, resources, and future opportunities. Learning also takes place during a formative developmental period and is organized through sustained teacher and peer relationships. These characteristics justify an education-specific theory centered on the dignity, fairness, and developmental sustainability of learning.

The contribution of DLT therefore lies less in claiming that each individual construct is new than in specifying a new focal appraisal and organizing existing constructs into a testable explanatory sequence. Its distinctive propositions are that structural educational conditions and the relational school environment contribute to perceived decent learning partly through three proximal psychological mechanisms, and that perceived decent learning may subsequently contribute to functioning partly through motivational internalization. These propositions allow future research to examine whether perceived decent learning explains additional variance beyond school climate, psychological safety, belonging, learning motivation, school satisfaction, and academic stress.

DLT is not limited to students who belong to visibly disadvantaged groups. Social position may shape exposure to resource constraints, institutional marginalization, exclusionary relationships, and unsustainable learning demands, but similar problems may also occur in highly resourced and high-performing settings. The form and strength of the proposed pathways may therefore vary across educational systems, developmental stages, cultures, and social positions. The initial model provides a common structure for examining this variation without assuming that all students experience the same educational conditions or interpret them in the same way.

### Research implications

First, DLT requires systematic empirical examination of its full directional pathway. Initial studies should test whether structural educational conditions and the relational school environment are associated with learning safety, learning belonging, and meaning in learning; whether these three proximal psychological mechanisms are associated with perceived decent learning; and whether perceived decent learning is subsequently associated with motivational internalization and behavioral, psychological, and developmental functioning. Empirical models should examine the two proposed stages of mediation separately. In the first stage, learning safety, learning belonging, and meaning in learning are expected to partially mediate the relationships between contextual antecedents and perceived decent learning. In the second stage, motivational internalization is expected to partially mediate the relationships between perceived decent learning and the three functioning domains. The residual direct paths specified in Propositions 4 and 8 should be retained and compared rather than assuming complete mediation. Longitudinal research will be necessary to evaluate the proposed temporal ordering. Repeated assessments could examine whether changes in structural and relational conditions precede changes in the three proximal mechanisms and perceived decent learning and whether changes in perceived decent learning precede motivational internalization and subsequent functioning. Competing temporal models should also be examined because students' motivation and functioning may influence their later appraisals of learning conditions. Multilevel designs will be particularly important because institutional arrangements, classroom practices, and relational climates may operate at school or classroom levels, whereas psychological mechanisms and learning appraisals are primarily assessed at the student level. Such research would help determine which pathways are robust across levels and contexts and whether a more parsimonious version of the model can be supported without losing its central explanatory structure.

Second, DLT offers a new platform for integrating educational psychology and the sociology of education. Existing research on learning has often focused either on individual motivation, cognition, and emotion, or on educational stratification, institutional pressure, and resource distribution. The boundary between these lines of inquiry remains substantial. The value of DLT lies in refusing to treat structure and psychology as competing explanations. Instead, it treats them as distinct but interrelated levels that jointly shape students' perceptions of learning. Future research may use this framework to identify the combinations of structural conditions, relational environments, and proximal psychological mechanisms that are most strongly associated with perceived decent learning, thereby advancing interdisciplinary dialogue and methodological integration.

Third, perceived decent learning requires systematic measurement development and validation. Although numerous instruments assess school climate, belonging, psychological safety, learning motivation, engagement, and academic stress, measures specifically focused on students' appraisal of whether learning preserves dignity, operates through procedurally fair arrangements, and supports sustainable development remain limited. Future scale development should examine the dimensional structure of perceived decent learning and compare alternative models involving dignity preservation, procedural fairness, and developmental sustainability as related but distinguishable appraisal domains. Qualitative item development and content validation should be followed by exploratory and confirmatory factor analyses, comparisons of alternative measurement models, and tests of reliability, convergent validity, discriminant validity, and incremental validity. Particular attention should be given to distinguishing perceived decent learning from its antecedents and psychological mechanisms. Learning safety, learning belonging, meaning in learning, teacher support, peer inclusion, recognition, academic workload, and opportunity accessibility should not be treated as interchangeable indicators of the focal construct. Instead, their relationships with perceived decent learning should be examined through separate measures. Future research should also assess measurement invariance across developmental stages, social positions, educational settings, and cultural contexts. Longitudinal measurement invariance will be especially important for determining whether changes in perceived decent learning reflect substantive developmental change rather than changes in how students interpret the measurement items.

Fourth, cross-cultural and cross-stage research will be equally important for the further development of the theory. Although the present article focuses primarily on adolescent learning contexts, perceived decent learning is unlikely to be limited to secondary education. University students, vocational learners, and adult learners may also encounter evaluative pressure, relational exclusion, diminished meaning, and disrupted learning rhythms. Future research should therefore examine the applicability of DLT across educational stages and compare the mechanisms and manifestations of perceived decent learning in secondary education, higher education, and continuing education. Such work would help extend the explanatory boundaries of the theory.

### Practice implications

DLT may provide a framework for examining students' learning difficulties at individual, classroom, school, and policy levels. Because the theory remains to be empirically tested, these implications should be understood as proposed directions for assessment and intervention rather than as established effects.

At the individual level, DLT encourages educators, school mental health professionals, and counselors to examine learning difficulties beyond explanations based solely on ability, motivation, or self-discipline. Inattention, avoidance, procrastination, reduced participation, or school refusal may have multiple causes, including mental health difficulties, family circumstances, prior learning experiences, and educational conditions (Gubbels et al., 2019). Assessment may therefore consider whether students experience learning safety, learning belonging, and meaning in learning and whether they appraise the learning process as dignity-preserving, procedurally fair, and developmentally sustainable. These constructs should be assessed separately rather than inferred directly from students' behavior.

At the classroom and school levels, the framework directs attention to both structural and relational conditions. Structural review may include resource and opportunity access, evaluation and disciplinary arrangements, academic workload, scheduling, and opportunities for recovery. Relational review may include the manner in which teachers respond to mistakes, communicate expectations, deliver feedback, and recognize students' efforts, as well as whether peer interaction permits participation without persistent ridicule or exclusion. Such practices may contribute to learning safety, learning belonging, and meaning in learning, which may subsequently shape students' broader appraisal of learning.

DLT also suggests that support should not be limited to changing students' coping or motivation. Student-focused support may help students articulate learning difficulties, identify personally relevant meaning in learning, use available relational support, and develop adaptive responses to academic demands. At the same time, schools should examine whether institutional and relational arrangements are themselves contributing to humiliation, exclusion, procedural unfairness, or unsustainable learning demands. The relative effectiveness of individual- and environment-focused approaches should be evaluated empirically.

At the policy level, DLT raises questions about how educational quality is defined. Achievement, progression, efficiency, and participation remain important indicators, but they do not establish whether the learning process is experienced as decent. Educational policies may therefore also consider whether evaluation procedures are transparent and consistently applied, whether resources and opportunities are distributed accessibly, whether learning schedules permit adequate recovery and social participation, and whether institutional practices protect students from persistent devaluation or exclusion.

These implications do not suggest that demanding education is necessarily inconsistent with decent learning. Academic challenge may remain compatible with perceived decent learning when expectations are fairly organized, students are treated with dignity, and demands do not chronically undermine psychological functioning or longer-term development. Future intervention and policy research should examine whether changes in these conditions are associated with improvements in perceived decent learning and subsequent student functioning.

### Limitations and future directions

DLT remains a preliminary theory and is subject to several limitations. First, its initial formulation is primarily intended to explain adolescents' perceptions of formal schooling, particularly in settings characterized by sustained institutional demands, evaluative dependence, and limited freedom to leave or substantially alter the learning environment. The model should not yet be assumed to apply unchanged to early childhood education, higher education, vocational training, adult learning, or informal learning contexts. Research across educational stages is needed to determine which constructs and pathways remain stable and which require modification.

Second, the meaning and relative importance of the proposed constructs may vary across cultural and institutional contexts. Educational systems differ in curricular organization, evaluation practices, teacher authority, expectations of effort, and the balance between individual and collective educational goals (Tao and Hong, 2014; Tlili et al., 2023). These differences may influence how students understand dignity preservation, procedural fairness, and developmental sustainability, as well as how learning safety, learning belonging, and meaning in learning contribute to the formation of perceived decent learning. Cross-cultural research should therefore examine both measurement invariance and structural variation rather than assuming that the model operates identically across contexts.

The normative meaning of “decent” learning also requires attention during construct development. Future studies should not assume that dignity preservation, procedural fairness, and developmental sustainability have identical meanings across settings. Qualitative and participatory work with students and relevant educational stakeholders can be used to examine how these principles are understood and expressed in different educational contexts. Interviews, focus groups, participatory workshops, and cognitive interviewing may help identify culturally or developmentally specific interpretations before formal scale validation and cross-cultural comparison.

Third, the initial model is intentionally parsimonious and does not incorporate all factors that may influence students' perceptions of learning. Family relationships, personality characteristics, prior academic experiences, physical and mental health, and individual coping resources may affect how students encounter and interpret the same educational conditions. These factors may operate as covariates, moderators, competing explanations, or sources of reciprocal influence. Their exclusion from the initial model reflects the need for conceptual clarity rather than an assumption that they are unimportant.

Fourth, the directional pathways proposed in DLT have not yet been established empirically. Structural and relational conditions may influence students' psychological experiences and appraisals, but students' prior motivation, functioning, and behavior may also shape how teachers and peers respond to them and how they subsequently interpret educational conditions. Cross-sectional evidence cannot distinguish these possibilities. Longitudinal, multilevel, and mixed-method studies should therefore compare the proposed sequence with reciprocal and alternative temporal models.

Future research should test the full theoretical pathway rather than isolated associations. This includes relationships from structural educational conditions and the relational school environment to learning safety, learning belonging, and meaning in learning; from these proximal mechanisms to perceived decent learning; from perceived decent learning to motivational internalization; and from motivational internalization to behavioral, psychological, and developmental functioning. Residual direct pathways should also be examined to determine whether the proposed mediation processes are partial and whether their strength differs across developmental stages, social positions, educational systems, and cultural settings.

Measurement development remains central to this agenda. Studies should examine the dimensional structure of perceived decent learning, its distinction from contextual antecedents and proximal psychological mechanisms, and its incremental validity beyond school climate, psychological safety, belonging, motivation, academic stress, and school satisfaction. Longitudinal and cross-group measurement invariance will be necessary before mean differences or developmental changes can be interpreted confidently.

DLT should therefore be regarded as an open but bounded theory. Future extensions should be guided by evidence that an additional construct improves explanatory value without reproducing existing mechanisms or weakening conceptual parsimony. Through cumulative empirical testing, the theory may be refined, simplified, or revised in response to evidence concerning its construct boundaries, temporal ordering, and applicability across educational contexts.

The core claims of DLT should also be open to falsification. The theory would be challenged if perceived decent learning cannot be empirically distinguished from adjacent constructs such as school climate, psychological safety, belonging, motivation, academic stress, or school satisfaction. Its proposed contribution would also be weakened if perceived decent learning provides no incremental explanatory value beyond these constructs or if longitudinal studies consistently fail to support the proposed ordering from contextual conditions to proximal psychological mechanisms, perceived decent learning, motivational internalization, and subsequent functioning. Such findings would require revision or narrowing of the theory rather than expansion of the model to accommodate unsupported pathways.

## Conclusion

This article proposes DLT as a preliminary explanation of how adolescents form overall appraisals of the quality of learning through structural and psychological processes. DLT shifts attention beyond achievement, motivation, and individual ability by asking whether learning is experienced as preserving dignity, operating through procedurally fair arrangements, and supporting sustainable development.

The initial model organizes this explanation into a directional but empirically testable sequence. Structural educational conditions and the relational school environment are proposed to shape learning safety, learning belonging, and meaning in learning. These proximal psychological mechanisms may contribute to perceived decent learning, which may subsequently be associated with motivational internalization and with behavioral, psychological, and developmental functioning. The model also allows for residual direct relationships rather than assuming complete mediation. The distinctive contribution of DLT lies in positioning perceived decent learning as an appraisal level between educational conditions and subsequent motivational and developmental processes. The theory does not claim that its individual components are entirely new. Instead, it integrates structural, relational, psychological, and motivational perspectives around the specific question of whether learning is organized and experienced as a dignified and developmentally sustainable human activity. DLT should therefore be regarded as an open but bounded theoretical proposal rather than as an empirically established causal model. Future longitudinal, multilevel, cross-cultural, and measurement research is needed to test its construct boundaries, temporal ordering, incremental validity, and applicability across educational contexts. Such work will determine whether perceived decent learning provides a useful additional explanation of how educational environments become connected with students' motivation, functioning, and longer-term development.

## Data Availability

The original contributions presented in the study are included in the article/supplementary material, further inquiries can be directed to the corresponding author.

## References

[B1] Abren G. T. Negasi R. D. Abebe A. S. (2025). Exploring the mediating role of dimensions of academic task engagement in the relationship among student-teacher relationship, academic task value, and positive development outcomes. Cogent Psychol. 12:2547781. doi: 10.1080/23311908.2025.2547781 PMC12755818 41474697

[B2] Allen K.-A. Slaten C. Hong S. Lan M. Craig H. May F. . (2024). Belonging in higher education: a twenty 20 year systematic review. J. Univ. Teach. Learn. Pract. 21, 1–55. doi: 10.53761/s2he6n66

[B3] Allotey E. García-Carrión R. Villardón-Gallego L. Soler-Gallart M. (2023). Transforming the educational experiences of marginalized students in Ghana through dialogic literary gatherings. Human. Soc. Sci. Commun. 10:313. doi: 10.1057/s41599-023-01801-z

[B4] Ansari A. Hofkens T. L. Pianta R. C. (2020). Teacher-student relationships across the first seven years of education and adolescent outcomes. J. Appl. Dev. Psychol. 71:101200. doi: 10.1016/j.appdev.2020.101200

[B5] Astor R. A. Noguera P. Fergus E. Gadsden V. Benbenishty R. (2021). A call for the conceptual integration of opportunity structures within school safety research. School Psychol. Rev. 50, 172–190. doi: 10.1080/2372966X.2020.1854621

[B6] Baumeister R. F. Leary M. R. (1995). The need to belong: Desire for interpersonal attachments as a fundamental human motivation. Psychol. Bull. 117, 497–529. doi: 10.1037/0033-2909.117.3.497 7777651

[B7] Beal S. J. Crockett L. J. (2010). Adolescents' occupational and educational aspirations and expectations: links to high school activities and adult educational attainment. Dev. Psychol. 46, 258–265. doi: 10.1037/a0017416 20053022 PMC6379913

[B8] Benítez-Agudelo J. C. Restrepo D. Navarro-Jimenez E. Clemente-Suárez V. J. (2025). Longitudinal effects of stress in an academic context on psychological wellbeing, physiological markers, health behaviors, and academic performance in university students. BMC Psychol. 13:753. doi: 10.1186/s40359-025-03041-z 40629456 PMC12239435

[B9] Blustein D. L. Kenny M. E. Di Fabio A. Guichard J. (2019). Expanding the impact of the psychology of working: engaging psychology in the struggle for decent work and human rights. J. Career Assess. 27, 3–28. doi: 10.1177/1069072718774002

[B10] Brachtl S. Ipser C. Keser Aschenberger F. Oppl S. Oppl S. Pakoy E. K. . (2023). Physical home-learning environments of traditional and non-traditional students during the COVID pandemic: exploring the impact of learning space on students' motivation, stress and wellbeing. Smart Learn. Environ. 10:7. doi: 10.1186/s40561-023-00222-4 40477797 PMC9851902

[B11] Branje S. (2022). Adolescent identity development in context. Curr. Opin. Psychol. 45:101286. doi: 10.1016/j.copsyc.2021.11.006 35008027

[B12] Branje S. de Moor E. L. Spitzer J. Becht A. I. (2021). Dynamics of identity development in adolescence: a decade in review. J. Res. Adolesc. 31, 908–927. doi: 10.1111/jora.12678 34820948 PMC9298910

[B13] Branje S. Morris A. S. (2021). The impact of the COVID-19 pandemic on adolescent emotional, social, and academic adjustment. J. Res. Adolesc. 31, 486–499. doi: 10.1111/jora.12668 34448306 PMC8646893

[B14] Brännlund A. Edlund J. (2020). Educational achievement and poor mental health in Sweden: the role of family socioeconomic resources. Educ. Inq. 11, 69–87. doi: 10.1080/20004508.2019.1687079

[B15] Bronfenbrenner U. (2000). “Ecological systems theory,” in Encyclopedia of Psychology, Vol. 3, ed. A. E. Kazdin (Washington, DC: American Psychological Association), 129–133. doi: 10.1037/10518-046

[B16] Cashman M. Strandh M. Högberg B. (2023). Have performance-based educational reforms increased adolescent school-pressure in Sweden? A synthetic control approach. Int. J. Educ. Dev. 103:102922. doi: 10.1016/j.ijedudev.2023.102922

[B17] Chen P. L. Lin C. H. Lin I. H. Lo C. O. (2023). The mediating effects of psychological capital and academic self-efficacy on learning outcomes of college freshmen. Psychol. Rep. 126, 2489–2510. doi: 10.1177/00332941221077026 35343336

[B18] Chin C. (2006). Classroom interaction in science: Teacher questioning and feedback to students' responses. Int. J. Sci. Educ. 28, 1315–1346. doi: 10.1080/09500690600621100

[B19] Cole E. R. (2009). Intersectionality and research in psychology. Am. Psychol. 64, 170–180. doi: 10.1037/a0014564 19348518

[B20] Cook D. A. Artino A. R. Jr. (2016). Motivation to learn: an overview of contemporary theories. Med. Educ. 50, 997–1014. doi: 10.1111/medu.13074 27628718 PMC5113774

[B21] Decker A. L. Leonard J. Romeo R. Itiat J. Hubbard N. A. Bauer C. C. C. . (2025). Exploration is associated with socioeconomic disparities in learning and academic achievement in adolescence. Nat. Commun. 16:6342. doi: 10.1038/s41467-025-61746-6 40634312 PMC12241540

[B22] DeOrtentiis P. S. Van Iddekinge C. H. Wanberg C. R. (2022). Different starting lines, different finish times: the role of social class in the job search process. J. Appl. Psychol. 107, 444–457. doi: 10.1037/apl0000915 33998822

[B23] Do K. T. Paolizzi S. G. Hallquist M. N. (2024). How adolescents learn to build social bonds: a developmental computational account of social explore-exploit decision-making. Dev. Cogn. Neurosci. 69:101415. doi: 10.1016/j.dcn.2024.101415 39089173 PMC11342119

[B24] Downing V. R. Cooper K. M. Cala J. M. Gin L. E. Brownell S. E. (2020). Fear of negative evaluation and student anxiety in community college active-learning science courses. CBE—Life Sci. Educ. 19:ar20. doi: 10.1187/cbe.19-09-0186 32453679 PMC8697658

[B25] Duffy R. D. Autin K. L. Bott E. M. (2015). Work volition and job satisfaction: examining the role of work meaning and person–environment fit. Career Dev. Q. 63, 126–140. doi: 10.1002/cdq.12009

[B26] Duffy R. D. Blustein D. L. Diemer M. A. Autin K. L. (2016). The psychology of working theory. J. Couns. Psychol. 63, 127–148. doi: 10.1037/cou0000140 26937788

[B27] Evans M. Boucher A. R. (2015). Optimizing the power of choice: supporting student autonomy to foster motivation and engagement in learning. Mind Brain Educ. 9, 87–91. doi: 10.1111/mbe.12073

[B28] Fernández-Sogorb A. Sanmartín R. Vicent M. Gonzálvez C. (2021). Identifying profiles of anxiety in late childhood and exploring their relationship with school-based distress. Int. J. Environ. Res. Public Health 18:948. doi: 10.3390/ijerph18030948 33499079 PMC7908635

[B29] Fidan A. Koçak Usluel Y. (2024). Emotions, metacognition and online learning readiness are powerful predictors of online student engagement: a moderated mediation analysis. Educ. Inform. Technol. 29, 459–481. doi: 10.1007/s10639-023-12259-6

[B30] García-Almeida D. Cabrera-Nuez M. T. (2020). The influence of knowledge recipients' proactivity on knowledge construction in cooperative learning experiences. Act. Learn. High. Educ. 21, 79–92. doi: 10.1177/1469787418754569

[B31] Gkiouleka A. Huijts T. Beckfield J. Bambra C. (2018). Understanding the micro and macro politics of health: Inequalities, intersectionality and institutions—a research agenda. Soc. Sci. Med. 200, 92–98. doi: 10.1016/j.socscimed.2018.01.025 29421476

[B32] Gonda D. Tirpáková A. Pavlovičová G. Duriš V. (2024). The role of a team psychological safety feeling in teamwork in the classroom. Heliyon 10:e37618. doi: 10.1016/j.heliyon.2024.e37618 39309768 PMC11415665

[B33] Grieve R. Woodley J. Hunt S. E. McKay A. (2021). Student fears of oral presentations and public speaking in higher education: a qualitative survey. J. Furth. High. Educ. 45, 1281–1293. doi: 10.1080/0309877X.2021.1948509

[B34] Gubbels J. van der Put C. E. Assink M. (2019). Risk factors for school absenteeism and dropout: a meta-analytic review. J. Youth Adolesc. 48, 1637–1667. doi: 10.1007/s10964-019-01072-5 31312979 PMC6732159

[B35] Hamel N. Schwab S. Wahl S. (2022). Social participation of German students with and without a migration background. J. Child Fam. Stud. 31, 1184–1195. doi: 10.1007/s10826-022-02262-9

[B36] Hanselman P. Domina T. Hwang N. (2021). Educational inequality regimes amid algebra-for-all: the provision and allocation of expanding educational opportunities. Soc. Forc. 100, 1722–1751. doi: 10.1093/sf/soab052 35935035 PMC9355462

[B37] Harrison C. J. Könings K. D. Dannefer E. F. Schuwirth L. W. T. Wass V. van der Vleuten C. P. M. (2016). Factors influencing students' receptivity to formative feedback emerging from different assessment cultures. Perspect. Med. Educ. 5, 276–284. doi: 10.1007/S40037-016-0297-X 27650373 PMC5035283

[B38] Heilman M. E. Caleo S. Manzi F. (2024). Women at work: pathways from gender stereotypes to gender bias and discrimination. Annu. Rev. Organ. Psychol. Organ. Behav. 11, 165–192. doi: 10.1146/annurev-orgpsych-110721-034105

[B39] Howard J. L. Bureau J. Guay F. Chong J. X. Y. Ryan R. M. (2021). Student motivation and associated outcomes: a meta-analysis from self-determination theory. Perspect. Psychol. Sci. 16, 1300–1323. doi: 10.1177/1745691620966789 33593153

[B40] Hughes C. Devine R. T. (2015). Individual differences in theory of mind from preschool to adolescence: achievements and directions. Child Dev. Perspect. 9, 149–153. doi: 10.1111/cdep.12124

[B41] Inouye K. Lee S. Oldac Y. I. (2023). A systematic review of student agency in international higher education. High. Educ. 86, 891–911. doi: 10.1007/s10734-022-00952-3 36439326 PMC9676751

[B42] Jacobsen B. S. (2025). Educational performance pressure and mental ill-being: the case of Danish primary and lower secondary schools, 1975–2024. Educ. Rev. 77, 2127–2144. doi: 10.1080/00131911.2024.2383897

[B43] Jain, S. K., Beers, N., Padrez, R., and Council on School, Health. (2024). School suspension and expulsion: policy statement. Pediatrics 154:e2024068466. doi: 10.1542/peds.2024-068466 39349407

[B44] Jang H. (2008). Supporting students' motivation, engagement, and learning during an uninteresting activity. J. Educ. Psychol. 100, 798–811. doi: 10.1037/a0012841

[B45] Jia M. Cheng J. (2024). Effect of teacher social support on students' emotions and learning engagement: a U.S.-Chinese classroom investigation. Human. Soc. Sci. Commun. 11:158. doi: 10.1057/s41599-024-02634-0

[B46] Kajastus K. Haravuori H. Kiviruusu O. Marttunen M. Ranta K. (2024). Associations of generalized anxiety and social anxiety with perceived difficulties in school in the adolescent general population. J. Adolesc. 96, 291–304. doi: 10.1002/jad.12275 37985185

[B47] Kilday J. E. Ryan A. M. (2025). Connecting engagement to classroom friendships and popular peers' prosocial behaviour. Br. J. Educ. Psychol. 95, 1080–1099. doi: 10.1111/bjep.12770 40131318

[B48] King R. B. McInerney D. M. Ganotice F. A. Villarosa J. B. (2015). Positive affect catalyzes academic engagement: cross-sectional, longitudinal, and experimental evidence. Learn. Individ. Differ. 39, 64–72. doi: 10.1016/j.lindif.2015.03.005

[B49] Korpershoek H. Canrinus E. T. Fokkens-Bruinsma M. de Boer H. (2020). The relationships between school belonging and students' motivational, social-emotional, behavioural, and academic outcomes in secondary education: a meta-analytic review. Res. Papers Educ. 35, 641–680. doi: 10.1080/02671522.2019.1615116

[B50] Košir K. Jugović I. P. (2025). Investigating the interplay between teacher-student and peer relationships in predicting academic engagement in early adolescents. Psychol. Sch. 62, 2608–2619. doi: 10.1002/pits.23491

[B51] Laranjeira M. Teixeira M. O. (2024). Examining teacher feedback, perceived competencies, interests, and achievement in fourth-grade students in Portugal. J. Career Dev. 51, 675–695. doi: 10.1177/08948453241290779

[B52] Li A. Yang D. D. Beauquesne A. Moro M. R. Falissard B. Benoit L. (2024). Somatic symptoms in school refusal: a qualitative study among children, adolescents, and their parents during the COVID-19 pandemic. Europ. Child Adolesc. Psychiatry 33, 2243–2251. doi: 10.1007/s00787-023-02313-6 37821562

[B53] Li J. Xue E. (2023). Dynamic interaction between student learning behaviour and learning environment: meta-analysis of student engagement and its influencing factors. Behav. Sci. 13:59. doi: 10.3390/bs13010059 36661631 PMC9855184

[B54] Liu A. Wei Y. Xiu Q. Yao H. Liu J. (2023). How learning time allocation make sense on secondary school students' academic performance: a Chinese evidence based on PISA 2018. Behav. Sci. 13:237. doi: 10.3390/bs13030237 36975262 PMC10045325

[B55] Maluenda-Albornoz J. Berríos-Riquelme J. Infante-Villagrán V. Lobos-Peña K. (2023). Perceived social support and engagement in first-year students: the mediating role of belonging during COVID-19. Sustainability 15:597. doi: 10.3390/su15010597

[B56] Marginson S. (2016). The worldwide trend to high participation higher education: dynamics of social stratification in inclusive systems. High. Educ. 72, 413–434. doi: 10.1007/s10734-016-0016-x

[B57] Martela F. Steger M. F. (2016). The three meanings of meaning in life: distinguishing coherence, purpose, and significance. J. Posit. Psychol. 11, 531–545. doi: 10.1080/17439760.2015.1137623

[B58] Mejía-Rodríguez A. M. Kyriakides L. (2022). What matters for student learning outcomes? A systematic review of studies exploring system-level factors of educational effectiveness. Rev. Educ. 10:e3374. doi: 10.1002/rev3.3374

[B59] Montes A. (2024). Transforming oneself: delving into the processes of habitus transformation in the higher education field. Br. J. Sociol. Educ. 45, 691–707. doi: 10.1080/01425692.2024.2355173

[B60] Mowat J. G. (2015). Towards a new conceptualisation of marginalisation. Europ. Educ. Res. J. 14, 454–476. doi: 10.1177/1474904115589864

[B61] Mundy L. K. Canterford L. Moreno-Betancur M. Hoq M. Viner R. M. Bayer J. K. . (2023). Learning outcomes in primary school children with emotional problems: a prospective cohort study. Child Adolesc. Ment. Health 28, 377–384. doi: 10.1111/camh.12607 36400427

[B62] Narayan R. Rodriguez C. Araujo J. Shaqlaih A. Moss G. (2013). “Constructivism—constructivist learning theory,” in The Handbook of Educational Theories, eds. B. J. Irby, G. Brown, R. Lara-Alecio, and S. Jackson (Information Age Publishing), 169–183. doi: 10.1108/978-1-61735-867-820251018

[B63] Neugebauer P. Prediger S. (2023). Quality of teaching practices for all students: multilevel analysis of language-responsive teaching for robust understanding. Int. J. Sci. Math. Educ. 21, 811–834. doi: 10.1007/s10763-022-10274-6

[B64] Novak J. D. (2002). Meaningful learning: the essential factor for conceptual change in limited or inappropriate propositional hierarchies leading to empowerment of learners. Sci. Educ. 86, 548–571. doi: 10.1002/sce.10032

[B65] Nungu L. Mukama E. Nsabayezu E. (2023). Online collaborative learning and cognitive presence in mathematics and science education: case study of University of Rwanda, College of Education. Educ. Inform. Technol. 28, 10865–10884. doi: 10.1007/s10639-023-11607-w 36779197 PMC9905761

[B66] Pascoe M. C. Hetrick S. E. Parker A. G. (2020). The impact of stress on students in secondary school and higher education. Int. J. Adolesc. Youth 25, 104–112. doi: 10.1080/02673843.2019.1596823

[B67] Pérez-Jorge D. Boutaba-Alehyan M. González-Contreras A. I. Pérez-Pérez I. (2025). Examining the effects of academic stress on student wellbeing in higher education. Human. Soc. Sci. Commun. 12:449. doi: 10.1057/s41599-025-04698-y

[B68] Prananto K. Cahyadi S. Lubis F. Y. Hinduan Z. R. (2025). Perceived teacher support and student engagement among higher education students—a systematic literature review. BMC Psychol. 13:112. doi: 10.1186/s40359-025-02412-w 39934874 PMC11817619

[B69] Qin T. Wei P. Xie Y. (2025). The multi-level influence mechanism of family background on children's education level: the moderating effect of socioeconomic status. PLoS ONE 20:e0323477. doi: 10.1371/journal.pone.0323477 40367232 PMC12077803

[B70] Ran Y. Wu X. Wang Y. Zhou Z. Yin Y. (2025). Examining the after-school study burden of Chinese secondary school students following burden reduction policies: a sociological analysis. BMC Psychol. 13:918. doi: 10.1186/s40359-025-03199-6 40813696 PMC12355888

[B71] Roscigno V. J. Tomaskovic-Devey D. Crowley M. (2006). Education and the inequalities of place. Soc. For. 84, 2121–2145. doi: 10.1353/sof.2006.0108

[B72] Rothers A. Cohrs J. C. (2023). What makes people feel respected? Toward an integrative psychology of social worth. Psychol. Rev. 130, 242–259. doi: 10.1037/rev0000393 36227285

[B73] Ryan R. M. Deci E. L. (2000). Self-determination theory and the facilitation of intrinsic motivation, social development, and wellbeing. Am. Psychol. 55, 68–78. doi: 10.1037/0003-066X.55.1.68 11392867

[B74] Ryan R. M. Deci E. L. (2020). Intrinsic and extrinsic motivation from a self-determination theory perspective: definitions, theory, practices, and future directions. Contemp. Educ. Psychol. 61:101860. doi: 10.1016/j.cedpsych.2020.101860

[B75] Saracho O. N. (2023). Theories of child development and their impact on early childhood education and care. Early Child. Educ. J. 51, 15–30. doi: 10.1007/s10643-021-01271-5

[B76] Schnitzler K. Holzberger D. Seidel T. (2021). All better than being disengaged: student engagement patterns and their relations to academic self-concept and achievement. Europ. J. Psychol. Educ. 36, 627–652. doi: 10.1007/s10212-020-00500-6

[B77] Semeraro C. Giofrè D. Coppola G. Lucangeli D. Cassibba R. (2020). The role of cognitive and non-cognitive factors in mathematics achievement: the importance of the quality of the student-teacher relationship in middle school. PLoS ONE 15:e0231381. doi: 10.1371/journal.pone.0231381 32310988 PMC7170247

[B78] Seo E. Clapper M. Hecht C. A. Crosnoe R. Yeager D. S. (2025). Peer mindset culture as a developmental context for belonging. Educ. Psychol. Rev. 37:103. doi: 10.1007/s10648-025-10082-8 41180316 PMC12575459

[B79] Soudien C. (2020). Complexities of difference and their significance for managing inequality in learning: lessons from the COVID-19 crisis. PROSPECTS 49, 59–67. doi: 10.1007/s11125-020-09486-x 32836428 PMC7333787

[B80] Stern E. (2017). Individual differences in the learning potential of human beings. npj Sci. Learn. 2:2. doi: 10.1038/s41539-016-0003-0 30631449 PMC6220331

[B81] Tao V. Y. K. Hong Y.-y. (2014). When academic achievement is an obligation: perspectives from social-oriented achievement motivation. J. Cross Cult. Psychol. 45, 110–136. doi: 10.1177/0022022113490072

[B82] Tarabini A. Jacovkis J. Montes A. (2018). Factors in educational exclusion: including the voice of the youth. J. Youth Stud. 21, 836–851. doi: 10.1080/13676261.2017.1420765

[B83] Terrin É. Triventi M. (2023). The effect of school tracking on student achievement and inequality: a meta-analysis. Rev. Educ. Res. 93, 236–274. doi: 10.3102/00346543221100850

[B84] Thiem K. C. Dasgupta N. (2022). From precollege to career: barriers facing historically marginalized students and evidence-based solutions. Soc. Issues Policy Rev. 16, 212–251. doi: 10.1111/sipr.12085

[B85] Tlili A. Wang H. Gao B. Shi Y. Zhiying N. Looi C.-K. . (2023). Impact of cultural diversity on students' learning behavioral patterns in open and online courses: a lag sequential analysis approach. Int. Learn. Environ. 31, 3951–3970. doi: 10.1080/10494820.2021.1946565

[B86] Trinidad J. E. (2024). An organizational sociology of education: using structural, network, and ecological perspectives to study schools. Sociol. Inq. 94, 968–993. doi: 10.1111/soin.12583

[B87] Tumuheki P. B. Zeelen J. Openjuru G. L. (2024). Towards a transformative lifelong learning agenda for non-traditional students at university. J. Adult. Contin. Education 30, 22–38. doi: 10.1177/14779714231196044

[B88] Urhahne D. Wijnia L. (2023). Theories of motivation in education: an integrative framework. Educ. Psychol. Rev. 35:45. doi: 10.1007/s10648-023-09767-9

[B89] Valiente C. Swanson J. DeLay D. Fraser A. M. Parker J. H. (2020). Emotion-related socialization in the classroom: considering the roles of teachers, peers, and the classroom context. Dev. Psychol. 56, 578–594. doi: 10.1037/dev0000863 32077726 PMC7041856

[B90] van Balen J. Gosen M. N. de Vries S. Koole T. (2022). “What do you think?” How interaction unfolds following opinion-seeking questions and implications for encouraging subjectification in education. Linguist. Educ. 69:101037. doi: 10.1016/j.linged.2022.101037

[B91] Wang J. Hazari Z. (2018). Promoting high school students' physics identity through explicit and implicit recognition. Phys. Rev. Phys. Educ. Res. 14:020111. doi: 10.1103/PhysRevPhysEducRes.14.020111

[B92] Wang M.-T. Degol J. L. (2016). School climate: a review of the construct, measurement, and impact on student outcomes. Educ. Psychol. Rev. 28, 315–352. doi: 10.1007/s10648-015-9319-1

[B93] Wang M.-T. Eccles J. S. (2012). Social support matters: longitudinal effects of social support on three dimensions of school engagement from middle to high school. Child Dev. 83, 877–895. doi: 10.1111/j.1467-8624.2012.01745.x 22506836

[B94] Wansink B. G.-J. Mol H. Kortekaas J. Mainhard T. (2023). Discussing controversial issues in the classroom: exploring students' safety perceptions and their willingness to participate. Teach. Teach. Educ. 125:104044. doi: 10.1016/j.tate.2023.104044

[B95] Wentzel K. R. Muenks K. McNeish D. Russell S. (2017). Peer and teacher supports in relation to motivation and effort: a multi-level study. Contemp. Educ. Psychol. 49, 32–45. doi: 10.1016/j.cedpsych.2016.11.002

[B96] Wigfield A. Eccles J. S. (2000). Expectancy–value theory of achievement motivation. Contemp. Educ. Psychol. 25, 68–81. doi: 10.1006/ceps.1999.1015 10620382

[B97] Wong M. D. Dosanjh K. K. Jackson N. J. Rünger D. Dudovitz R. N. (2021). The longitudinal relationship of school climate with adolescent social and emotional health. BMC Public Health 21:207. doi: 10.1186/s12889-021-10245-6 33485308 PMC7825179

[B98] Zengilowski A. Maqbool I. Deka S. P. Niebaum J. C. Placido D. Katz B. . (2023). Overemphasizing individual differences and overlooking systemic factors reinforces educational inequality. npj Sci. Learn. 8:13. doi: 10.1038/s41539-023-00164-z 37156826 PMC10166032

[B99] Zulaika G. Bulbarelli M. Nyothach E. van Eijk A. Mason L. Fwaya E. . (2022). Impact of COVID-19 lockdowns on adolescent pregnancy and school dropout among secondary schoolgirls in Kenya. BMJ Global Health 7:e007666. doi: 10.1136/bmjgh-2021-007666 35027438 PMC8761596

